# The Adaptive Behavior of a Soccer Team: An Entropy-Based Analysis

**DOI:** 10.3390/e20100758

**Published:** 2018-10-02

**Authors:** Yair Neuman, Navot Israeli, Dan Vilenchik, Yochai Cohen

**Affiliations:** 1 Department of Cognitive and Brain Sciences, Zlotowski Center for Neuroscience, Ben-Gurion University of the Negev, 84105 Beer-Sheva, Israel; 2 Wales Ltd., 52522 Ramat-Gan, Israel; 3 Department of Communication Systems Engineering, Ben-Gurion University of the Negev, 84105 Beer-Sheva, Israel; 4 Gilasio Coding, 6458701 Tel-Aviv, Israel

**Keywords:** organization/disorganization, team’s performance, soccer, entropy

## Abstract

To optimize its performance, a competitive team, such as a soccer team, must maintain a delicate balance between organization and disorganization. On the one hand, the team should maintain organized patterns of behavior to maximize the cooperation between its members. On the other hand, the team’s behavior should be disordered enough to mislead its opponent and to maintain enough degrees of freedom. In this paper, we have analyzed this dynamic in the context of soccer games and examined whether it is correlated with the team’s performance. We measured the organization associated with the behavior of a soccer team through the Tsallis entropy of ball passes between the players. Analyzing data taken from the English Premier League (2015/2016), we show that the team’s position at the end of the season is correlated with the team’s entropy as measured with a super-additive entropy index. Moreover, the entropy score of a team significantly contributes to the prediction of the team’s position at the end of the season beyond the prediction gained by the team’s position at the end of the previous season.

## 1. Introduction

A competitive team must maintain a delicate balance between organization and disorganization. To illustrate this point, we may use the behavior of a soccer team. A soccer team is a complex social system (e.g., [1]), whose performance crucially dependents on its ability to coordinate the behavior of its members in such a way that goals are scored and the opponents’ attempts to score are blocked. One major aspect of the team’s behavior concerns *ball passes* between the players. While the positions and roles of players in modern football are loosely defined, the *general* schematic path of moving the ball is forward, from the first line of defense through the midfield, where most of the passes take place, and up to the attack by players (e.g., a goal poacher) who aim to score. 

The fact that most ball passes take place at the midfield is almost self-evident as this is the major medium through which the ball moves from the team’s first line to the forward players. This pattern can be easily observed in sites of soccer statistics. For example, analyzing the Premier League players’ ranking in terms of ball possessions/passes (http://www2.squawka.com, from August 2017), we can see that the top players are the midfielders/defenders.

Passing the ball is a highly complex and coordinated activity that must maintain an optimal balance between organization/disorganization, order/disorder, and certainty/uncertainty. In this sense, it is a specific instance of a well-documented dynamics of complex adaptive systems (e.g., [2]). 

Knowing who is passing to who means reducing the entropy of ball passes to maximize communication and cooperation between the team’s players. For example, knowing that the probability of a pass from player A to player M is higher than the probability of a pass from player A to all other players, may strongly support the team’s coordinated behavior when A is holding the ball. As such, minimizing the entropy of passes may support the team’s coordinated behavior but it might: (1) limit its *degrees of freedom* in moving the ball and *surprising* its opponent; and (2) expose the team to counter attacks by the competing team that may try, for instance, to block player M when it observes player A moving the ball. The price of maximizing the certainty of passes might be an expected behavior and vulnerability to the opponent’s attacks. In other words, the performance of a soccer team may be modeled through the entropy of its ball-passes. 

**Figure 1:**
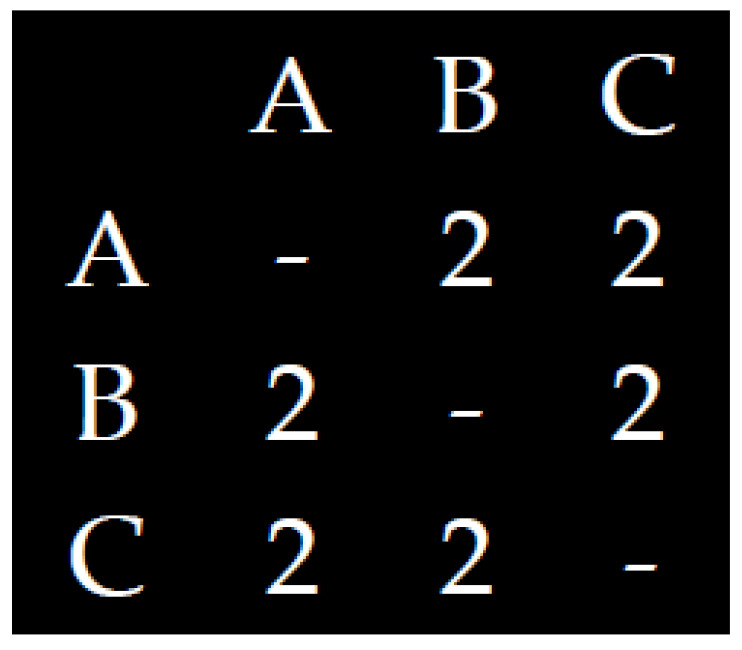
A distribution of ball passes.

**Figure 2:**
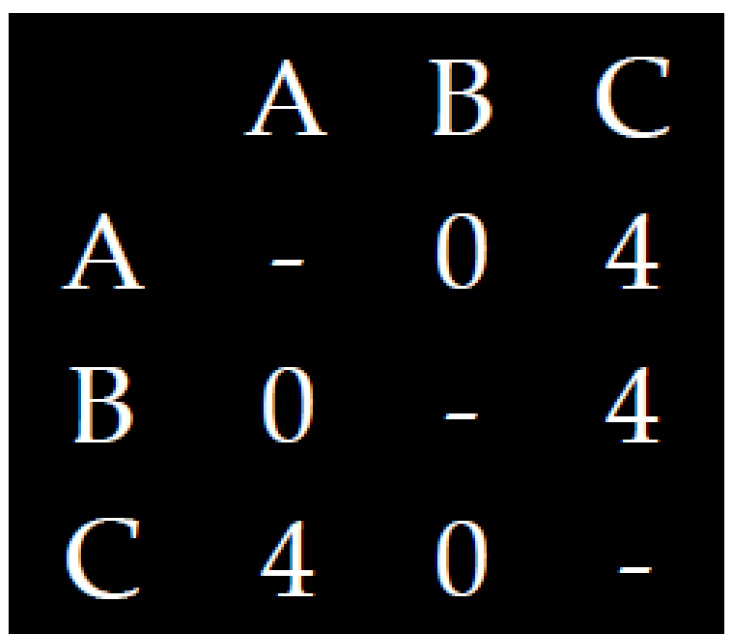
A more heterogenous distribution of ball passes.

The entropy of ball passes may be simply considered as the degree of heterogeneity (i.e., uniformity)/homogeneity (i.e., variability) that characterizes the ball passes between the players. The closer to uniformity is the distribution of ball passes between the players, the more uncertain we are who is going to get the ball. For example, Figure 1 and Figure 2 present extreme cases of uniformity and variability, of high and low entropy of ball basses between three players:

Taking this competitive perspective into account, we may understand that, on the one hand, a successful team may strive to minimize its entropy in order to maximize communication. On the other hand, the team may strive to maximize its entropy in order to maximize its degrees of freedom and prevent from its opponent to respond to an ordered pattern of behavior. In fact, this idea perfectly resonates with the Interaction Performance Theory [3] from which we may hypothesize that the performance of a soccer team is influenced by the opponent team. While O’Donoghue [3] presented a general theory pointing to the adaptive nature of a player, he did not measure the performance of players in terms of entropy. In the context of the current study, we measured the performance of a soccer team in terms of ball-passes entropy and the way this measure of performance is adapted to the behavior of the opponent team. In this sense, our paper seems to provide supporting evidence for the Interacting Performance Theory albeit from the perspective of entropy analysis. 

It must be noted that the ball passing behavior of a soccer team has already been studied and we do not pretend to be the first to do it. For example, Goncalves et al. [4] applied networks measures (e.g., betweenness centrality) to the passing behavior of 44 Portuguese players. However, their aims, procedure and measures were quite different from those of the current study. The same is true for the study of Ric et al. [5] where ball passes of 19 players have been analyzed for the aim of studying the way in which the players’ spatial restrictions influenced their exploratory tactical behavior. These studies are different from the current paper in which the ball passes of soccer teams are modeled using a unique kind of parameterized entropy (i.e., Tsallis entropy) to examine the way a competitive team manages the optimal balance between organization and disorganization. It must be emphasized that our major interest is not in better understanding the specific dynamic of soccer but to examine the general dynamic of complex competitive and adaptive systems in the unique context of soccer. Emphasizing this point, we may return to our main hypothesis. 

It seems that maintaining an optimal balance between organization and disorganization is a challenge facing a competitive team in general and the success of addressing this challenge may be correlated with the team’s performance. This hypothesis is firmly grounded in several classical ideas regarding the modeling of complex systems, such as in Ashby’s Law of Requisite Variety [6]. The concept of variety may be used to describe the total number of the system’s possible states. In our case, the distribution of ball passes between the players. Ashby’s proposed that there must be an optimal correspondence between the system’s variety of actions and the variability of the systems it models. In our case, a competitive soccer team should produce enough variability of ball passes in order to counter attack the “modeling” attempts of its opponent seeking to identify patterns of ball passes. This idea is clearly expressed in the dynamics between within-group cooperation and conflict in various contexts [7] and in some other deep theoretical ideas such as Jaynes Principle of Maximum Entropy [8] proposing that an optimal modeling of a system’s distribution should involve choosing the distribution that maximizes the entropy of the distribution under certain constraints. For example, let us assume that one observes the behavior of a soccer team and notice that on average player A passes the ball six times to player B. Modeling the behavior of player A, one should try to reconstruct the distribution of ball passes while assuming that this distribution has an average of six. However, there are several possible ball passes distributions with an average of six, so the choice is very difficult. Jaynes [8] proposed that, among all potential distributions, under the constraint of an average equals six, we should choose the one with the maximum entropy, i.e., the one with the max variability. The deeper reason for this proposal is that, by maximizing the variability of this distribution, we maximize our ability to model the system, as proposed by Ashby [6], and maximize our ability to account for uncertainty and surprise. The same deep theoretical logic may hold for competitive teams striving to maximize their performance. When facing its opponent, the team may strive to maximize the entropy of its behavior in order to prevent from the opponent to gain information and learn patterns of its behavior. However, for its inside communication process, the team should strive to minimize the entropy in order to maximize communication and performance. 

In this paper, we have studied the interplay between maximizing/minimizing entropy in the context of soccer games and hypothesized that the success of a soccer team is correlated with its ability to produce ordered patterns of ball passes and to maintain the above discussed balance between order and disorder. Therefore, our first hypothesis was that the entropy of the ball passes should be correlated with the team’s success at the end of the season. 

## 2. Materials and Methods 

### 2.1. Data

The English Premier League is the most watched sport league in the world. It involves twenty soccer clubs competing each season. During a season, each club/team plays against the others twice for a total of 38 games in a season. We purchased from Perform group the data of the 2015/2016 season, describing ball passes between the players of each team. The data were provided for each game at the team’s level of analysis. 

### 2.2. Procedure

For each team, we formed a matrix of ball passes between the players, summing the passes between each pair of players over the entire season. Next, we computed the entropy of the matrix using Tsallis entropy [9,10] defined as:

(1) $$Sq\left(pi\right)=\frac{1}{q-1}(1-\sum pi^{q})$$

where in our case pi describes the probability of ball passes between each pair of players (excluding “self” passes) and the entropy is computed across all players in a team. *q* is a real number parameter known as the Tsallis or the entropy index. 

The reason for choosing Tsallis entropy was that this specific form of parameterized entropy has been designed to analyze systems where correlations exist between its micro-states, and therefore it seemed specifically relevant for analyzing the data of soccer games. This point is elaborated and illustrated in the following sections. Meanwhile, let us see a simple illustration of the entropy index. Let us assume that we would like to compute the ball passes entropy of player A who passed the ball four times to player B and only twice to player C. If we compute the entropy of the ball passes using entropy index of 2, then the entropy is approximately 0.45, but if we use an entropy index of 0.2 then the entropy is approximately double: 0.9. Using the entropy index of 0.2 has given much more weight to the relatively rare event of passing the ball to player C. It has “amplified” rare events that in some contexts may be of high interest, as illustrated below.

For the first analysis, we used entropy index of 0.2, as this super-additive index amplifies “rare” events [9], which, in our case, for instance, include ball passes where forward players are involved. As ball passes involving forward players are relatively rare, *amplifying* such rare events gives them extra-weight in the final entropy score and may have consequence for identifying patterns in the behavior of a soccer team that may be correlated with a team’s success, as shown in the next sections. 

## 3. Results

In this section, we present several subsections of analysis that aim to promote our understanding regarding the relation between ball-passes entropy and the team’s performance. Each section ends with the main conclusion and, at the end of the three rounds of analysis, we present the main results gained across the three rounds.

### 3.1. Analysis 1

The aim of the first analysis was to use the entropy measure of the team to predict its *position* at the end of the season, where the highest rated team is ranked “1” and the lowest rated team “20”.

Two points must be clarified. First, we use the term “prediction” in the sense of *curve fitting*, and not in the sense of *forecasting*. The current study does not aim to support the field of sport predictive analytics. Second, it must be noted that in contrast with other games such as basketball, the prediction of a soccer game/team performance is extremely difficult as goals are rare and a direct association between team’s measures and performance is therefore hard to establish [11]. This point is specifically acute for the current study where the performance of a single season is analyzed with only twenty teams.

**Figure 3:**
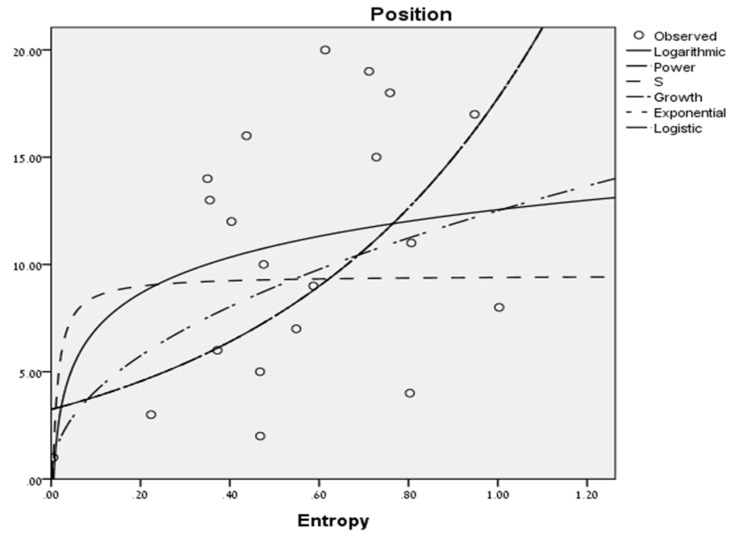
Regression models predicting the team’s position.

We first performed our analysis with two parameters of the Tsallis entropy index (*q* = 0.2 and *q* = 2) and with Shannon’s entropy (*q* = 1), as these measures represent: (1) the common Shannon entropy measure; (2) a super-additive entropy index (*q* < 1) that amplifies the probability of rarer events; and (3) a sub-additive entropy index (*q* > 1) that amplifies the probability of common events. Figure 3 presents the different regression models that were found to significantly predict position:

The best prediction of position using the normalized entropy measures of the ball passes (0–1) was gained through a power regression model with the entropy index of 0.2 (F(1, 18) = 14.49, *p* < 0.001, R^2^ = 0.446). In terms of Cohen’s norms, as used in the social sciences [12] where the dynamics of teams is usually studied, the effect size expressed by this R Square result is considered to be “high”. The best regression models, with an entropy index of 2 and the Shannon entropy, gained less successful predictions (R^2^ = 0.229 and R^2^ = 0.293, respectively). 

In sum, *it was found that the higher is the entropy of the team, the lower is its position at the end of the season*. This finding may be easily explained as a higher entropy score means that the pattern of the group’s ball passes is less organized and a less organized team is a less successful team.

One may argue that the correlation between position and the entropy measure may be explained by the team’s possession of the ball; the more time the team has to possess the ball, as reflected in its overall number of ball passes, the more time it has to organize itself. Therefore, successful teams who hold the ball for more time express a lower level of entropy. Indeed, the group’s overall number of ball passes was found to be significantly correlated with position (*r* = −0.52, *p* = 0.009); the better was the team’s rank at the end of the season, the more ball passes it produced through the season. However, the correlation between the entropy measure and the team’s position remained significant even when controlling for ball passes (*r* = 0.552, *p* = 0.007).

Main conclusion: The higher is the entropy of the team, the lower is its position at the end of the season.

#### Analysis using Singular Value Decomposition (SVD)

We have previously shown that the team’s level of order, as expressed by the entropy of its ball passes matrix, is correlated with the team’s performance. Another way of examining the same behavior may be to measure the “simplicity” of the ball passes matrix through SVD. Hence, the following analysis aims to support the validity of the previous findings, this time by using another measure of organization.

Every matrix $X\;\in R^{m\times n}$ can be written as the product

(2) $$X={\mathrm{USV}}^{T}$$

where $U\;\in \;R^{m\times m}$ and $V\;\in \;R^{n\times n}$ are orthogonal matrices and S is an m×n diagonal matrix with non-negative entries, ordered in the following way:

$$\sigma_{1}\geq \sigma_{2}\cdots \sigma_{n}\geq 0$$

The columns of U and V are called the left and right singular vectors, respectively, and the $\sigma_{i}^{\prime }s$ are the singular values. An easy corollary of the above equation is the fact that each row $r_{i}$ of X can be written as a linear combination of the columns of $U=\left(u_{1},u_{2},\ldots ,u_{m}\right)$

(3a) $$r_{i}=\overset{m}{\underset{k=1}{\sum }}v_{ik}\sigma_{k}u_{k}$$

Similarly, the columns of X may be written as

(3b) $$c_{j}=\overset{m}{\underset{k=1}{\sum }}u_{jk}\sigma_{k}v_{k}$$

This idea can be applied to our soccer data. Let $X_{t}^{\left(k\right)}$ be a matrix whose rows and columns are indexed by the n players of team number t. The ${\left(i,j\right)}^{\mathrm{th}}$ entry of $X_{t}^{\left(k\right)}$ is the total number of balls that player i passed to player j in game number k. Player i is therefore characterized by an n-dimensional vector $r_{i}$ which details the balls it passed to every other player of the team. All the game matrices in our dataset were nearly full rank. In other words, each row $r_{i}$ of $X_{t}^{\left(k\right)}$ is a linear combination of (n−1) or (n−2) basis vectors. An instructive way to think of the basis vectors is as if each basis vector is a virtual player, and the vector describes the balls that this virtual player passed to all the real players of the team. In this terminology, we may represent player i as the weighted sum of the virtual player pattern of interaction. Now, let us look at an SVD decomposition of a game matrix X and suppose for the sake of argument that only $\sigma_{1}$ and $\sigma_{2}$ are non-zero. 

From Equation (3a) we may write each $r_{i}$ as

$$r_{i}=v_{i1}\sigma_{1}u_{1}+v_{i2}\sigma_{2}u_{2}.$$

In other words, the balls-passes profile of every player is a linear combination of merely two virtual players. Similarly, the balls-received profile of every player, $c_{j},$ is also a linear combination of just two vectors.

$$c_{j}=u_{i1}\sigma_{1}v_{1}+u_{i2}\sigma_{2}v_{2}.$$

In reality, it is not true that $\sigma_{3}=\sigma_{4}=$ … =$\sigma_{n}=0$ (otherwise, the game matrix would have had rank two and not nearly full rank), however if $\sigma_{1}\gg \sigma_{3}$, then there exists an ε that depends on the ration $\sigma_{1}/\sigma_{3}$ such that for all players i,

$$\|r_{i}-(v_{i1}\sigma_{1}u_{1}+v_{i2}\sigma_{2}u_{2)}\|\leq \varepsilon$$

**Figure 4:**
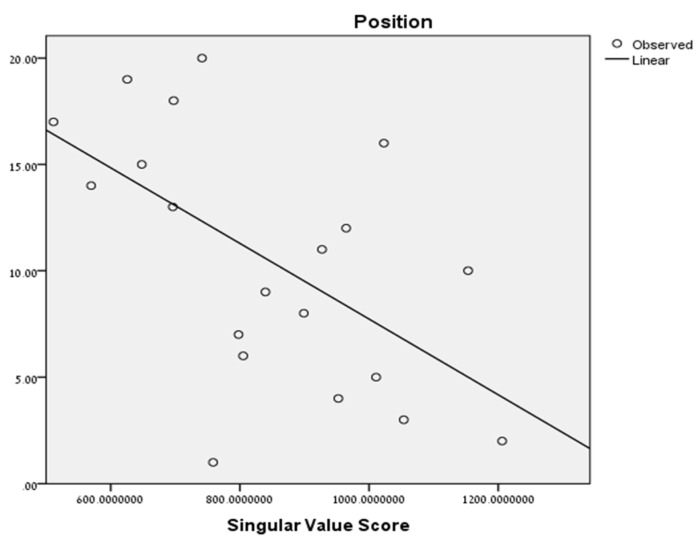
The SVD prediction of position.

In other words, we may approximate the pattern of each player with only two virtual players. The quality of the approximation depends on the ratio $\sigma_{1}/\sigma_{3}$—the larger it is, the better approximation we gain. Therefore, our working hypothesis was that the fewer virtual players needed to obtain a good approximation of the team, the higher the in-team certainty. To test this hypothesis, we computed the singular value of each team’s ball passes matrix and used it to predict position in a linear regression model. Figure 4 presents the curve fit of the model:

The regression model was found to be statistically significant (F(1, 18) = 9.04, *p* = 0.008, with R^2^ = 0.33), and as such supports our first hypothesis regarding the team’s level of organization and its correlation with the team’s performance.

In sum, two tentative conclusions may be drawn from the first analysis. First, the higher is the entropy of the team’s general pattern of ball passes, the lower is its position at the final league table. Second, the relative success of the entropy measure with *q* = 0.2 may suggest that, when *privileging rare events in the ball pass matrix*, which is precisely the meaning of the super-additive index, our prediction improves. This hypothesis may hint to the role of certain type of players, such as the forward players who have a lower degree of ball passes, in gaining the team’s position. 

### 3.2. Analysis 2

As most ball passes are conducted in the midfield by midfield players, a reasonable hypothesis is that the rare events, privileged by the super-additive Tsallis index we chose (*q* = 0.2), those events supporting the prediction of the team’s position, are associated with the defenders and/or forward players. 

If the team’s performance, as measured through the Tsallis entropy, is “privileged” through the more ordered behavior of the forward players, then it should be expressed in the ability of the Tsallis entropy to predict the goals *scored* by the group. On the other hand, if the source of the success more heavily leans on the defenders’ behavior, then we should see it while trying to predict the goals *conceded* by the team. We tested this second hypothesis. 

We measured the correlation between the entropy measure with Tsallis index of 0.2 and goals scored/conceded by each team. Using Spearman’s rho, a positive and statistically significant correlation was found between the entropy measure and goals conceded (*r_s_* =0.49, *p* = 0.01). No correlation was found with goals scored. 

**Figure 5:**
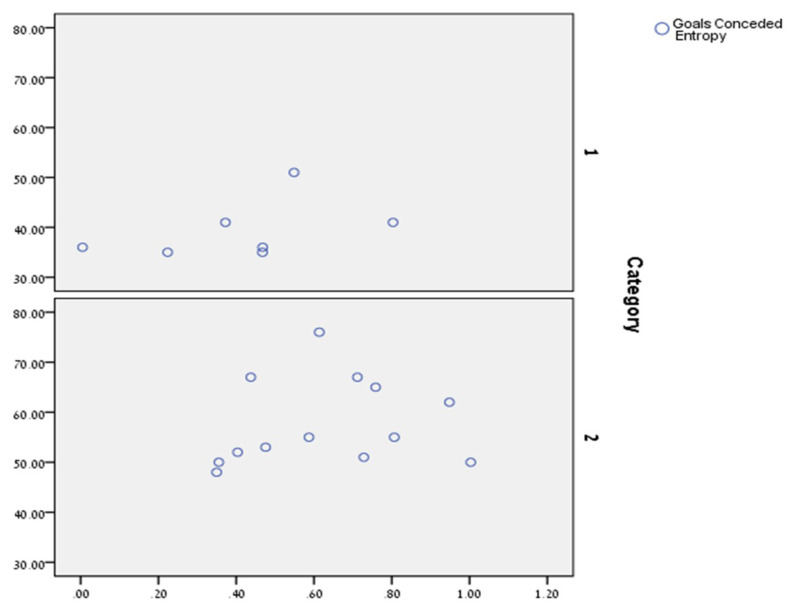
Overlay scatter for the two categories. The X-axis signifies the entropy and the Y-axis, goals conceded.

We may have another perspective of these findings, by dividing the teams into two categories: (1) the top seven teams that have gained some qualification, such as a qualification to participate in the Champions League; and (2) the rest of the teams. Figure 5 presents the overlay scatter of goals conceded by entropy for the two categories:

Through this scatter, one may visualize the abovementioned result. The above results may point to the important role of the defenders in coordinating the ball’s movement. It is reasonable to hypothesize that defenders who are more coordinated in moving the ball forward are more coordinated in defending their goalkeeper from an approaching attack. While the praise in soccer is usually given to those who score goals, our analysis may point to an interesting pattern which is the important weight of the defenders’ “negative” success in avoiding goals. While this pattern may be trivial to football fans, our empirical analysis shows how important is this trivial pattern. This result may also point to the *importance of using Tsallis entropy* that by amplifying certain micro states through the super-additive entropy index, may help us to better understand a soccer group as a complex social system by exposing patterns that cannot be identified through other measures of organization.

Main conclusion: It seems that the coordination between the ball passes of the defenders has a significant role in the team’s success.

### 3.3. Analysis 3

Beyond the ability to predict the team’s position using the entropy measure, we should recall that one of the most trivial predictors of the team’s final position may be the team’s position at the end of the previous season. For instance, using a linear regression with the team’s position at the previous season as a predictor, it was possible to significantly predict its current position (F(1, 15) = 10.24, *p* = 0.006), with R^2^ = 0.40. In this context, it is interesting to measure the relative contribution of the entropy measure to the prediction of the team’s position beyond the one gained by the previous season’s position. When adding the entropy measure to the linear regression model that includes the previous year position, the prediction has been significantly increased (F(2,14) = 9.45, *p* = 0.003, with R^2^ = 0.57) with a roughly 17% of improvement in the explained variance. This result points to the importance of the team’s entropy in improving the prediction of the team’s final position at the league. 

Main conclusion: The entropy of the ball passes has a significant contribution for predicting the team’s position beyond the predictive value that may be gained through the team’s past success.

### 3.4. Analysis 4

The results presented so far suggest that the team’s dynamics as measured through the entropy of passes is correlated with the team’s performance. However, this analysis does not *directly* address the *competitive adaptive* aspect of the game. Although the entropy score of the matrix probably represent to some extent the adaptive behavior of a team in response to its opponent, it does not *directly* measure the adaptive aspect of the team’s behavior. 

To address this challenge, we measured the entropy of each team during every game and compared it to the entropy of its opponent’s team at the specific game. An important point that must be emphasized is that a team that has a higher level of entropy than its opponent is not necessarily the weaker team. For example, a well-organized team may hold the ball for a longer time and produce more ball passes that indicate its dominance in the game. In such a case, its higher entropy level does not indicate a lower level of organization but the existence of more degrees of freedom in moving the ball as a complementary aspect of the team’s organization and dominance. 

**Figure 6:**
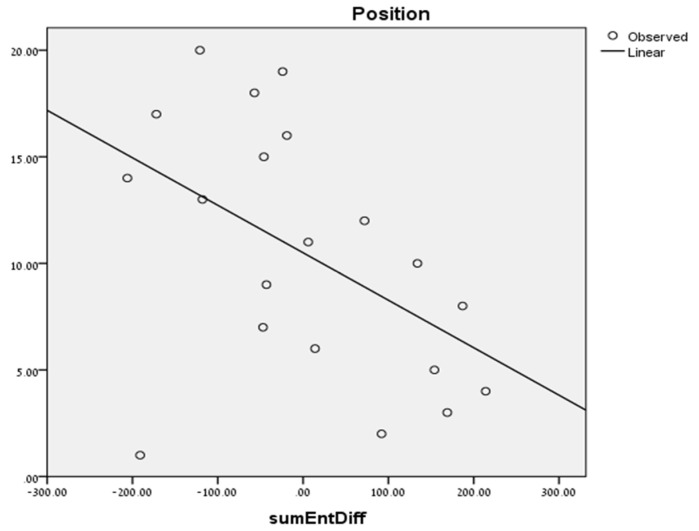
The linear regression line fitted to the correlation between sum of entropy differences and the team’s position.

We calculated the difference in entropy between each two competing teams in a specific match, and for each team computed the sum of entropy differences across the season. Figure 6 presents the linear regression line using the sum of entropy differences to predict the team’s position.

We can see that the higher is the *relative* entropy of a team per game, the better is its position. This finding is elaborated below. 

After computing the average entropy difference of a team, we computed the ratio between this score and the entropy measure of the team’s ball passes matrix (both normalized to 0–1 through the MinMax transformation), and applied log transformation to the outcome of this normalization to support linearity. 

The next hypothesis is that the new score—ENdif—will be correlated with the team’s success as a team expressing a higher level of relative entropy actually express more degrees of freedom in moving the ball and expresses a higher level of adaptive organization. 

ENdif predicted position in a linear regression model (F(1,17) = 22, *p* < 0.001, with R^2^ = 0.57); the higher is the entropy of the team in comparison with the entropy of its opponent, the better it is positioned at the end of the season. However, this finding may be confounded with the amount of time in which the more successful team possessed the ball. For example, imagine a situation where a less successful team cannot pass the midfield. In this case, the ball passes might be extremely limited and the entropy relatively low. In this case, the “higher” entropy of the most successful team is not an indication of more degrees of freedom but just a simple artifact of the time it possessed the ball. When measuring the Pearson correlation between ENdif and the team’s overall number of ball passes, the correlation was not found to be statistically significant. When using both ENTdif and ball passes in the model, the predictive power of the model has been increased to R^2^ = 0.60 only. Therefore, the possession time of the team does not seem to be a crucial factor in rebutting our argument. 

Based on the abovementioned results, we built a new linear regression model that included, in addition to ENTdif, the log transformed normalized scores of: (1) previous year’s position; (2) Tsallis entropy; and (3) ball passes. A backward conditional linear regression model was found statistically significant with ENTdif as *the only significant predictor* (F(1,14) = 24, *p* < 0.001, with R^2^ = 0.63).

The above findings extend our understanding of the team’s behavior. In general, we found that a more organized team is a more successful team. However, the less organized is the behavior of the team in a specific game, *as compared with its opponent*, the less expected its behavior to the opponent, the higher are the team’s degrees of freedom and the higher are the chances for scoring goals and avoiding goals. 

This is a highly important point. We usually think of organization and disorganization as oppositions. However, here the competency of a soccer team may be expressed, even paradoxically one may say, in forming an ordered pattern of ball passes on the one hand, while within this order to express a relatively higher level of disorder that expresses its relatively higher degrees of freedom in moving the ball. 

From a technical perspective, it must be remembered that the ENTdif score of a team is normalized by computing the ratio between the entropy *difference* score and the general entropy measure of the *same* team. For example, let us assume that team E_1_ is much better than team E_2_. E_1_ is a more organized team and its entropy score on the general ball passes matrix is “1” in comparison with the less organized team that scored “10”. During a certain game, the teams meet and the entropy score of team E_1_ is e_1_ = 5 and the entropy score of team E_2_ is e_2_ = 1. In general, the entropy of E_1_ is lower than the entropy of E_2_, however at the specific game it presents a higher level of entropy. The entropy difference in favor of team E_1_ is “4” and divided by “1”, it produces a higher entropy score (i.e., “4”) than the entropy difference score of E_2_ which is divided by “10”. The more general conclusions of our findings are discussed in the next section. 

Main conclusion: The higher is the entropy of the team in comparison with the entropy of its opponent, the better it is positioned at the end of the season.

## 4. Discussion

The coordinated activity of a competitive team may be conceived as an attempt to balance between the level of organization and certainty necessary to facilitate the communication between the team’s members and the level of disorganization and uncertainty necessary to mislead its opponent. In this paper, we have shown that this general logic is probably evident in soccer, although analyzing the behavior of English soccer team in a single season necessarily limits the validity of our findings. Our general conclusion is that the entropy of the team plays a significant role in predicting its performance as hypothesized. We have reached several conclusions that, given the limited scope of a scientific study, should be modestly presented and examined. 

The conclusions are as follows:

1. The higher is the entropy of the team, the lower is its position at the end of the season.
2. It seems that the coordination between the ball passes of the defenders has a significant role in the team’s success.
3. The entropy of the ball passes has a significant contribution for predicting the team’s position beyond the predictive value that may be gained through the team’s past success.
4. The higher is the entropy of the team in comparison with the entropy of its opponent, the better it is positioned at the end of the season.

We may summarize our contribution as follows. First, we have shown that measuring the entropy of the ball passes produces a statistically significant model fit when trying to predict the team’s position. As the Tsallis entropy has been formed in physics to study systems with correlations between its microstates, this result is of no surprise. However, in the context of soccer, optimizing the predictive power of the entropy measure through the super additive entropy index, may provide us with the ability to identify the important role of the defenders. This result is important as it gives an uneven weight to the different agents in the system. When studying the coordinated behavior of a starling flock for instance, the behavior of each bird is equally weighted [13]. However, it may be the case that, when studying the behavior of small human teams such as a soccer team, where *distributed cognition* is in action, different weights should be given to different nodes of this dynamic network in order to reveal interesting patterns. 

Our second major contribution is showing that the team’s balance between organization and disorganization is an important predictor of its final position. This measure of balance was found to provide even better prediction of the team’s final position than the previous season’s ranking. 

While these two major contributions have a theoretical value, they may have an applied value as well by providing an additional *feature* for the forecasting of soccer matches. The measures that we have introduced in this paper may be used as features for improving the predictive power of machine learning classifiers. However, as our data are limited to a single season of the English Premier League, and as our paper does not focus on the contribution of such features to forecasting models, we leave the practical applications of our study to future research. In addition, we did not study the added value of the entropy measures within a wider predictive model that includes other features such as the financial budget of the team. Building a predictive model, such as the one presented by Constantinou and Fenton [14] is clearly beyond the scope of the current study. Whether the entropy measures presented in this study may contribute to such a predictive model is an open question that should be empirically addressed in other studies. We sum by pointing to some interesting directions in studying the way a team’s level of organization may be correlated with its performance but necessarily acknowledge the limitations on our study and its preliminary nature.
